# Case Report: A rare presentation of short posterior ciliary artery occlusion with paracentral acute middle maculopathy

**DOI:** 10.3389/fmed.2025.1638909

**Published:** 2025-07-15

**Authors:** Dai Yuanmin, Xu Jiehui

**Affiliations:** Department of Ophthalmology, Zhejiang Hospital, Hangzhou, China

**Keywords:** short posterior ciliary arteries, paracentral acute middle maculopathy, optical coherence tomography, fluorescein angiograph, ischemia

## Abstract

The occlusion of the short posterior ciliary arteries (SPCAs), a severe yet infrequent ocular vascular pathology, becomes particularly uncommon when it coexists with paracentral acute middle maculopathy (PAMM). We report a rare instance involving a middle-aged male who experienced an occlusion of the posterior ciliary short artery alongside paracentral acute middle maculopathy (PAMM). A 48-year-old male patient presented with a sudden loss of vision in the upper left visual field and a slight decrease in visual acuity. Multimodal imaging, including scanning laser ophthalmoscope (SLO) imaging, revealed a “tongue-shaped” region of normal retina with mild retinal edema both above and below the optic disk. Fluorescein angiography (FA) exhibited minor delays in arterial perfusion and venous reflux, and a “tongue-shaped” area of normal retina was observed, with delayed hypofluorescence in the retinal areas above and below the optic disk, coupled with diminished choroidal background fluorescence, forming a “triangle” that pointed toward the optic disk. Optical coherence tomography (OCT) revealed retinal edema and hyperreflective bands within the inner nuclear layer and mild thickening of the inner retina. PAMM arises from inner retinal vascular lesions leading to macular hypoperfusion; however, its occurrence in the context of choroidal ischemic diseases is seldom documented. Consequently, this case provides novel perspectives on the retinal blood supply system.

## Introduction

The posterior ciliary artery system, particularly the short posterior ciliary arteries (SPCAs), is of paramount importance in the supply of blood to the optic nerve head and the peripapillary choroid. The occlusion of these vessels commonly results in anterior ischemic optic neuropathy or choroidal infarction syndromes. Nevertheless, the anatomical variability in the distribution of SPCAs, with typically 2–5 branches supplying the temporal peripapillary choroid and nasal watershed zones, introduces the potential for atypical ischemic presentations when collateral circulation is compromised (1–3).

Paracentral acute middle maculopathy (PAMM), first characterized by Sarraf et al. (4), represents a distinct spectral-domain optical coherence tomography (SD-OCT) finding of hyperreflective band-like lesions at the inner nuclear layer (INL), reflecting ischemic injury to the intermediate and deep retinal capillary plexuses. While classically associated with retinal vascular disorders such as diabetic retinopathy and retinal vein occlusion (5), emerging evidence suggests that its pathophysiology may extend to broader choroidal vascular compromise (6).

They frequently manifest in individuals with predisposing factors for thrombus formation, geriatric patients, and those who have experienced invasive vascular procedures. Diagnosis can be established clinically through funduscopy, fluorescein angiography (FA), and optical coherence tomography (OCT) (4–6).

This report presents the first documented case of PAMM secondary to focal SPCA occlusion, confirmed through multimodal imaging. Offers a new perspective on the fundus manifestations when the inner retina is affected due to choroidal ischemia.

## Case presentation

**Figure 1:**
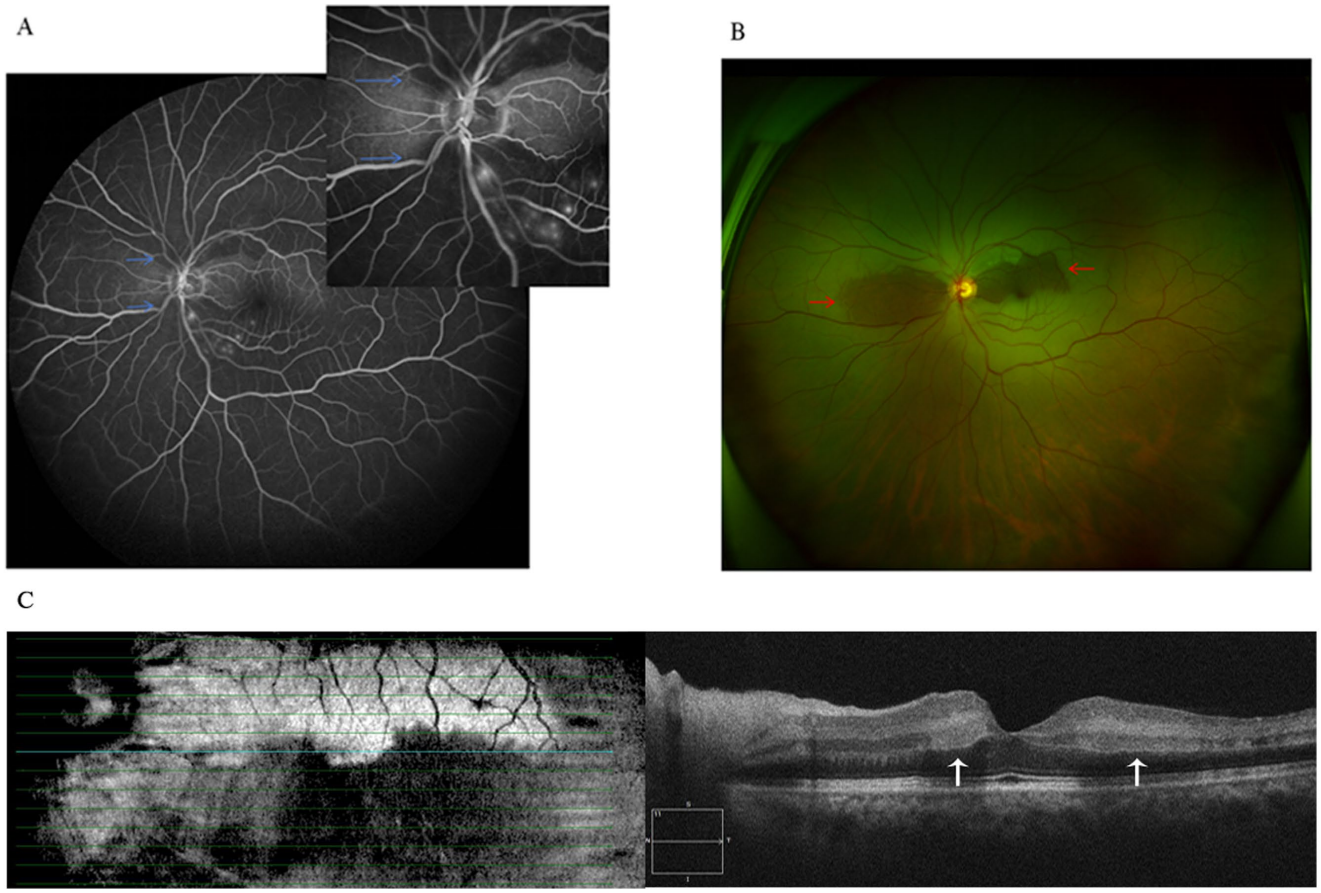
**(A)** Fluorescein angiography shows reduced choroidal background fluorescence except for the perfused areas (blue arrows). **(B)** Fundus photograph shows mild retinal edema in superior and inferior to the optic disc, while the perfused areas reveal a “tongue-shaped” region (red arrows). **(C)** Optical coherence tomography revealed retinal mild thickening with band of hyperreflectivity in the inner nuclear layer adjacent to the macula (white arrows).

**Figure 2:**
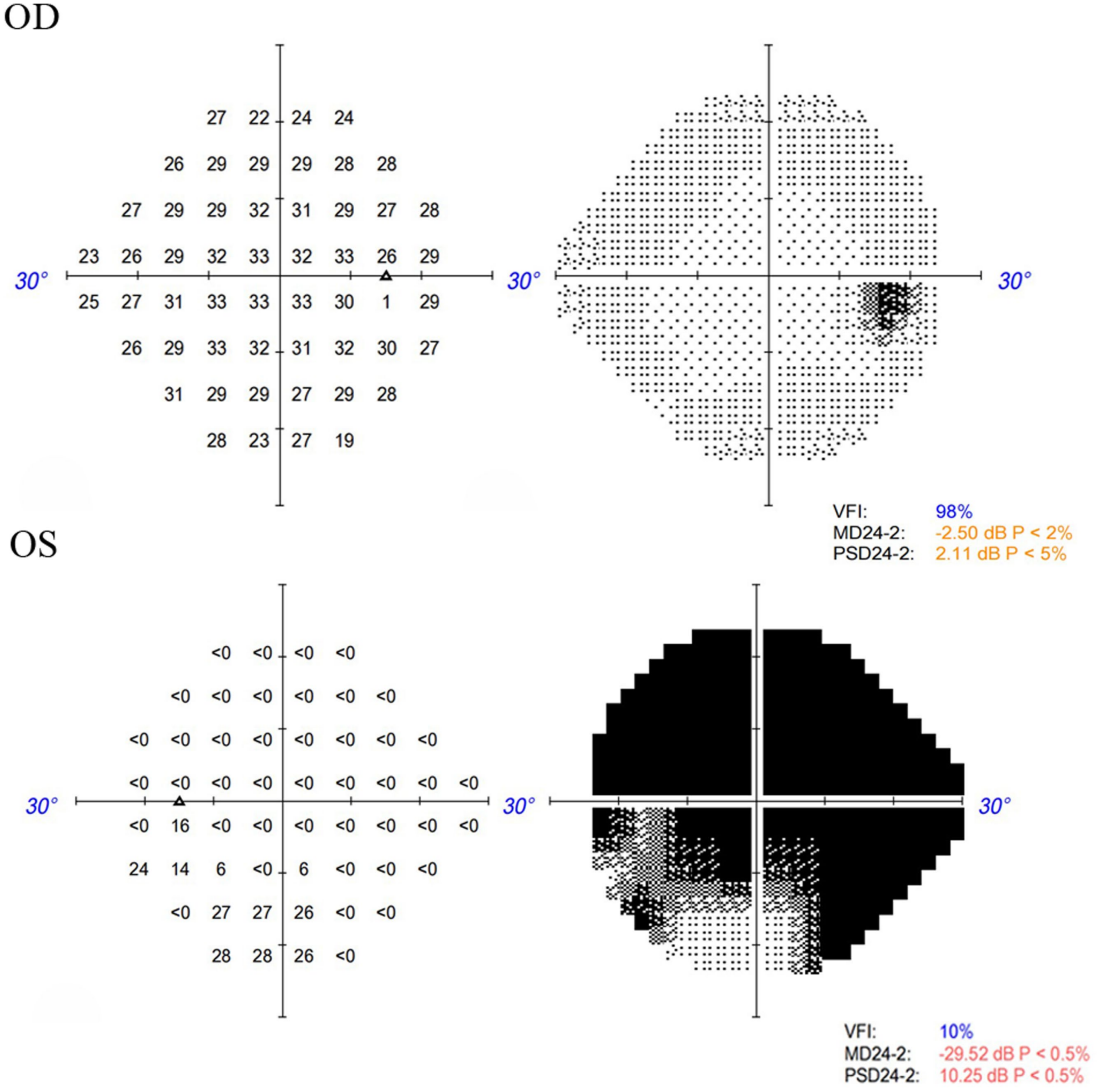
Visual field examination indicated a limited region of retained vision in the inferior nasal quadrant, accompanied by widespread visual field reduction in other areas in the left eye, and no significant defects in the right eye.

A 48-year-old male patient experienced a sudden loss of the left visual field, which persisted for half a day, and subsequently sought medical attention at another hospital. The patient’s medical record indicated a visual acuity of 20/63 in the left eye, and a provisional diagnosis of left central retinal artery occlusion was posited. Despite the administration of oral vasodilators, no amelioration of symptoms was observed. The following day, the patient presented to the outpatient department of our hospital. Ophthalmological examination revealed a visual acuity of 20/20 in the right eye and 20/63 in the left eye, with intraocular pressures measuring 14 mmHg in the right eye and 15 mmHg in the left eye. Additionally, the patient’s blood pressure was recorded at 115/75 mmHg. Notably, there was no pertinent family history, although the patient had a history of smoking. Slit-lamp examination of the anterior segment did not reveal any abnormalities. Funduscopic examination and scanning laser ophthalmoscope (SLO) imaging disclosed a “tongue-shaped” region of normal retina, with mild retinal edema located superiorly and inferiorly to the optic disk. Fluorescein angiography (FA) indicated an arm-to-retina circulation time of approximately 15 s, with minor delays in arterial filling and venous return. The imaging also exhibited a “tongue-shaped” area of normal retina, while the retinal regions above and below the optic disk displayed delayed hypofluorescence, forming a “triangle” that pointed toward the optic disk. A reduction in choroidal background fluorescence was noted, except in the perfused areas. Optical coherence tomography (OCT) revealed retinal edema and hyperreflective bands in the inner nuclear layer and mild thickening of the inner retina (Figure 1). The visual field examination of the left eye indicated a limited region of retained vision in the inferior nasal quadrant, accompanied by widespread visual field reduction in other areas, with a significant decline in visual field index (VFI) (Figure 2). The patient’s medical history comprised hypertrophic cardiomyopathy, which was being effectively managed through annual cardiological monitoring without the need for pharmacological treatment. An electrocardiogram (ECG) exhibited mild ST-segment alterations. Further systemic evaluations, including tests for antinuclear antibodies, antineutrophil cytoplasmic antibodies (ANCAs), blood viscosity, antiphospholipid syndrome, coagulation function, complete blood count, C-reactive protein (CRP), erythrocyte sedimentation rate (ESR), angiotensin-converting enzyme inhibitors (ACEIs), TORCH panel, thromboelastogram (TEG), transthoracic echocardiography (TTE), lower limb venous ultrasound, carotid ultrasound, brain magnetic resonance imaging (MRI), and thrombophilia gene screening, yielded no significant findings. Despite administering prompt treatment of medium-dose glucocorticoids, antiplatelet therapy, vasodilators, supplemental oxygen, and intraocular pressure-lowering treatments, the patient’s symptoms proved resistant to therapeutic interventions. This is primarily attributed to SPCAs being an end artery with an absence of collateral circulation. Once infarction occurs, the affected retinal tissue is unable to obtain sufficient blood flow from alternative vascular sources. Consequently, the ischemic damage in the affected region is largely irreversible.

## Discussion

The posterior ciliary artery (PCA), a branch of the ophthalmic artery, primarily supplies blood to the optic nerve, equatorial choroid, retinal pigment epithelium (RPE), outer retina, ciliary body (both inner and outer layers), and iris. Ischemic disturbances in the PCA circulation can lead to various ocular and optic nerve head (ONH) ischemic lesions, causing vision loss (5). According to Hehrey’s findings, no significant fundus changes occur within 1 h after PCA occlusion. However, 18 to 24 h later, fundus examination may reveal irregularly sized wedge-shaped or triangular white spots (7). Early-phase fluorescein angiography (FAG) shows delayed filling of the ciliary retinal artery and absent background choroidal fluorescence (7). Previous reports have linked choroidal vascular occlusion to subcutaneous injection of corticosteroid powder or cosmetic facial fillers. Most cases present with sudden, painless vision loss or visual field defects (8, 9).

In our case, the patient presented with an acute onset and moderate vision loss. SLO imaging revealed a “tongue-shaped” area of normal retina. FA indicated significant delay in choroidal blood flow perfusion but slight delays in arterial filling and venous return. OCT showed typical PAMM changes in the macula. Systemic evaluation was negative except for mild ST-segment changes on ECG. The patient had a history of smoking and hypertrophic cardiomyopathy, which was stable under cardiological surveillance. The main thrombotic risk factor here is the smoking history. Based on typical fundus changes and multi-modal imaging, the diagnosis is SPCAs with PAMM. PAMM, an ischemic change in the deep retinal vascular network, is often associated with central retinal vessel system ischemia. It is rarely reported in choroidal ischemic diseases, so this case offers new insights into the retinal blood supply system.

This case provides the first multimodal imaging evidence of isolated SPCA occlusion manifesting as acute PAMM, challenging the traditional compartmentalization between choroidal vascular insufficiency and retinal capillary ischemia. Recent advances in multimodal imaging have uncovered the paradoxical retinal manifestations of posterior ciliary ischemia. Based on prior studies, PAMM often occurs when inner retinal vascular lesions cause macular hypoperfusion (10, 11). In contrast, Balaratnasingam et al. demonstrated SPCA occlusion-induced deep capillary plexus ischemia (12). According to previous research, the main speculated causes of PAMM include hypoxia in the intermediate retinal tissue, watershed zone vulnerability, abnormality in capillary plexus dynamics, structural and microvascular vulnerability, and intermediate retinal layer susceptibility (9). These findings challenge the traditional dichotomy between choroidal and retinal vascular pathologies, suggesting a shared hemodynamic continuum.

Despite these insights, human reports of isolated SPCA occlusion presenting with isolated PAMM remain exceedingly rare. Current literature describes only three cases associated with PAMM with posterior ciliary pathology, all involving long posterior ciliary arteries or diffuse choroidal hypoperfusion (13–15). The unique anatomical trajectory of SPCAs penetrating the sclera of the optic nerve before arborizing in the peripapillary choroid raises critical questions about retrograde ischemic mechanisms affecting the middle retinal layers. Based on prior research by Yu et al., the capillaries within the macular region of primates exhibit a unique anatomical architecture, with spatiotemporal heterogeneity in the perfusion of their vascular networks (9, 16). Notably, the perifoveal area possesses a relatively thicker ganglion cell layer compared to other regions. Furthermore, the capillary plexus within the inner nuclear layer forms a ring-like structure composed of vertically or obliquely oriented vessels. Ischemia affecting this specific plexus manifests on OCT as a band-shaped region of hyperreflectivity, located within the outermost portion of the inner retinal layers. Additionally, ischemic alterations in the choroidal vasculature can induce secondary changes in this region due to disruptions in the oxygen gradient (16). The pathogenesis likely involves retrograde trans-choriocapillary ischemia, a mechanism diverging from prior models of PAMM development. While existing theories emphasize direct retinal arteriolar compromise or venous stasis (9), our patient’s intact central retinal artery and vein implicate choroidal vascular dynamics. The SPCAs’ unique anatomical course, penetrating the sclera 3–5 mm nasal to the optic nerve before branching into the Haller’s layer, creates a “vascular bottleneck.” Occlusion at this critical juncture (confirmed by ICGA filling defects) may disrupt pressure gradients across the choriocapillaris, impairing oxygen diffusion to the metabolically demanding INL region. This aligns with Yu’s experiments, which show that SPCA ligation reduces DCP oxygen saturation (12).

The primary limitation of this case report is that the initial assessment based on the patient’s fundus examination failed to consider SPCA occlusion. Following FA, the patient declined to undergo indocyanine green angiography (ICGA)—an examination that would have been particularly valuable for demonstrating choroidal ischemia. Future cases should prioritize completing this test to strengthen evidence. However, FA still provides strong evidence. This case shows that PAMM can occur in choroidal ischemic diseases.

## Conclusion

This first reported case of isolated SPCA occlusion manifesting as PAMM redefines our understanding of retinal–choroidal vascular interplay. The case ultimately illustrates that in the era of multimodal imaging, “retinal” ischemia may often be the scleral sentinel of deeper vascular compromise.

## Data Availability

The original contributions presented in the study are included in the article/supplementary material, further inquiries can be directed to the corresponding author.
